# Divergence of functions and expression patterns of soybean bZIP transcription factors

**DOI:** 10.3389/fpls.2023.1150363

**Published:** 2023-04-14

**Authors:** Lin Yue, Xinxin Pei, Fanjiang Kong, Lin Zhao, Xiaoya Lin

**Affiliations:** ^1^ Guangdong Provincial Key Laboratory of Plant Adaptation and Molecular Design, Guangzhou Key Laboratory of Crop Gene Editing, Innovative Center of Molecular Genetics and Evolution, School of Life Sciences, Guangzhou University, Guangzhou, China; ^2^ Key Laboratory of Soybean Biology of Ministry of Education China, Northeast Agricultural University, Harbin, China

**Keywords:** soybean, transcription factor, bZIP, function, expression patterns

## Abstract

Soybean (*Glycine max*) is a major protein and oil crop. Soybean basic region/leucine zipper (bZIP) transcription factors are involved in many regulatory pathways, including yield, stress responses, environmental signaling, and carbon-nitrogen balance. Here, we discuss the members of the soybean bZIP family and their classification: 161 members have been identified and clustered into 13 groups. Our review of the transcriptional regulation and functions of soybean bZIP members provides important information for future study of bZIP transcription factors and genetic resources for soybean breeding.

## Introduction

Transcription factors (TFs) can be grouped into different families according to their DNA-binding and multimerization domains. Basic region/leucine zipper (bZIP) TFs are characterized by a conserved bZIP domain composed of two motifs: a basic region responsible for binding to specific DNA sequences, and a leucine zipper motif required for dimerization (Hurst, 1995; Wingender et al., 2001; Jakoby et al., 2002). Plant bZIP TFs function in stress and hormone signaling, organ and tissue differentiation, photomorphogenesis, cell elongation, nitrogen/carbon balance, energy metabolism, flower development, seed development, pathogen defense, and gibberellin biosynthesis (Chern et al., 1996; Albani et al., 1997; Oyama et al., 1997; Fukazawa et al., 2000; Jakoby et al., 2002; Cluis et al., 2004; Corrêa et al., 2008; Weltmeier et al., 2009). The functions of plant bZIP proteins appear to be more complex and broader than those of other TFs (Wei et al., 2010).

Due to their crucial roles in numerous biological processes, bZIP TFs have been studied in many plant species: 78 bZIP genes have been identified in *Arabidopsis thaliana* (Dröge-Laser et al., 2018), 92 in rice (*Oryza sativa*) (Corrêa et al., 2008), 125 in maize (*Zea mays*) (Wei et al., 2012), 64 in cucumber (*Cucumis sativus*) (Yoshida et al., 2015a), and 69 in tomato (*Solanum lycopersicum*) (Li et al., 2015). Jakoby et al. (2002) classified *Arabidopsis* bZIP genes into 10 groups (A, B, C, D, E, F, G, H, I, and S) based on the similarity in the basic region and other conserved motifs. Dröge-Laser et al. (2018) reported four new members in *Arabidopsis*, AtbZIP76-AtbZIP79, and excluded AtbZIP73 as a pseudogene, and classified these 78 AtbZIPs into 13 groups (designated A-M). AtbZIPs are associated with a plethora of functions; most AtbZIPs in each group display group-specific properties (Jakoby et al., 2002; Dröge-Laser et al., 2018).

Soybean (*Glycine max* [L.] Merr) is an important food and industrial crop. Many bZIP genes have been found in soybean. Liao et al. (2008) identified 131 GmbZIP TFs and classified them into 10 groups (A, B, C, D, E, F, G, H, I, and S). Most GmbZIP proteins cluster with the AtbZIP proteins, whereas several GmbZIP members form a distinct S group. Wang et al. (2015) identified 138 GmbZIPs, and Zhang et al. (2018) identified and classified 160 GmbZIPs into 12 groups (A, B, C, D, E, F, G, H, I, J, K, and S). In soybean, 124 and 122 out of 160 GmbZIPs are involved in drought and flooding responses, respectively (Zhang et al., 2018), however, many GmbZIPs have been implicated in various biological processes besides abiotic stress responses. In this review, we analyze the cladistics and expression profiles of GmbZIP and AtbZIP genes, and focus on the well-studied GmbZIP genes, in order to summarize and predict the functions of GmbZIP TFs, and to provide perspectives for their further identification and use in soybean breeding.

## Cladistic analysis of GmbZIP proteins

**Figure 1:**
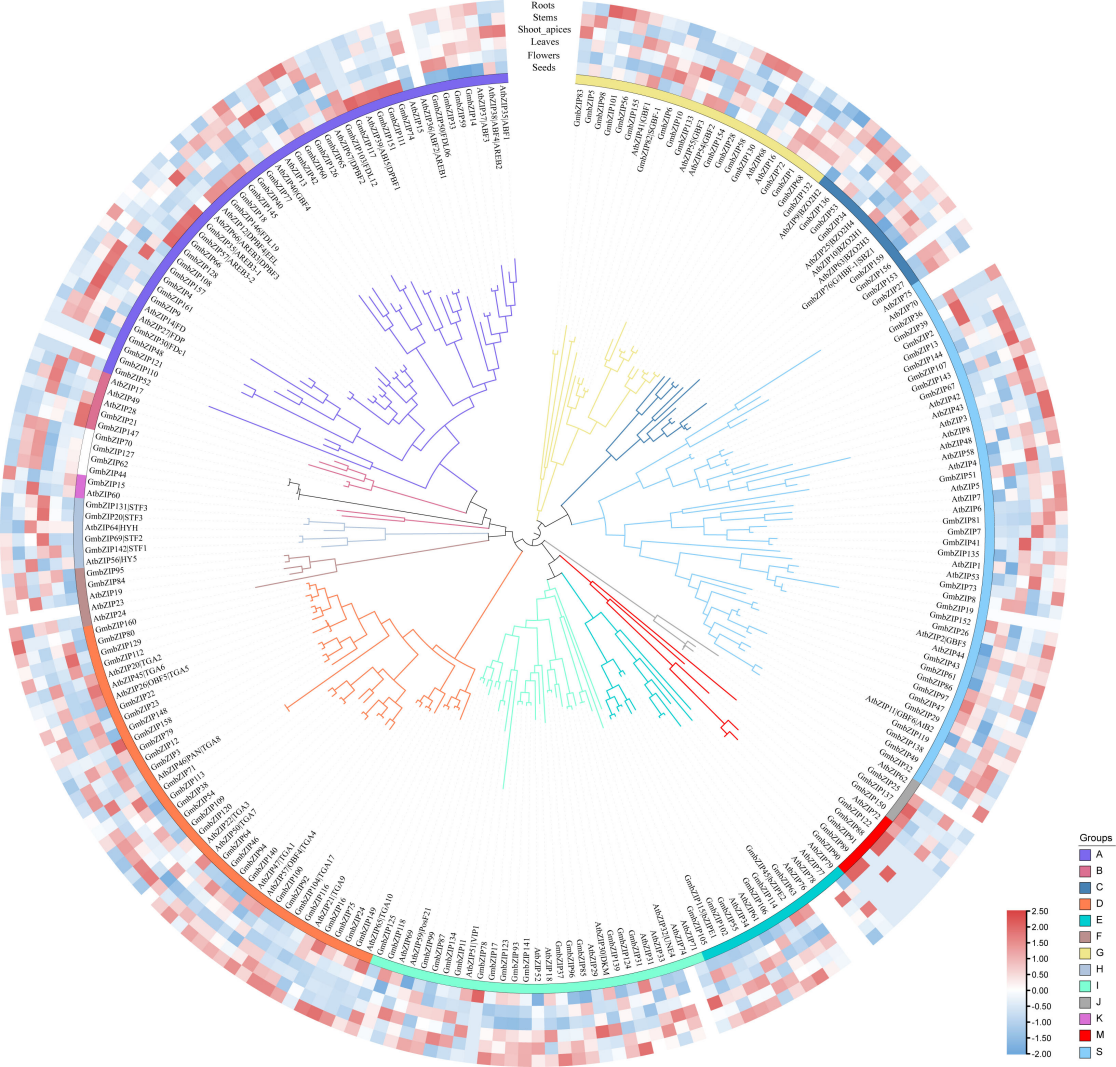
Phylogenetic analysis and expression profiles of soybean and Arabidopsis bZIP proteins.

Soybean and *Arabidopsis* bZIP proteins have been recently updated (Sussmilch et al., 2015; Dröge-Laser et al., 2018; Zhang et al., 2018), we regenerated the cladistic tree, and the names of soybean bZIP proteins were based on Zhang et al. (2018) in this review. We aligned the full-length GmbZIP and AtbZIP amino acid sequences by MAFFT v7.505 with default parameters and then conducted a cladistic analysis using iQtree with model JTT+F+R5 and polishing using iTOL (https://itol.embl.de). The GmbZIPs and AtbZIPs classified into 13 groups, which is the same as Zhang et al. (2018) and Dröge-Laser et al. (2018) (**Figure 1**).

## Expression patterns of GmbZIP genes

We obtained expression values (fragments per kilobase of exon per million mapped fragments (FPKMs)) of GmbZIP and AtbZIP genes in different tissues/organs (seeds, flowers, leaves, shoot apices, stems, and roots) from the websites at Phytozome12 (https://phytozome.jgi.doe.gov/pz/portal.html) (Schmutz et al., 2010), and AtGenExpress Plus-Extended Tissue Series in the Arabidopsis eFP Browser (http://bar.utoronto.ca/efp_arabidopsis/cgi-bin/efpWeb.cgi) with the Developmental Baseline as the parameter and other parameters remaining the default (Schmid et al., 2005) (**Figure 1**). *Arabidopsis* tissues/organs were selected for consisting with soybean tissues/organs. We removed *GmbZIP88*, *AtbZIP18*, *AtbZIP23*, *AtbZIP36*, *AtbZIP62*, *AtbZIP70*, *AtbZIP75*, and *AtbZIP76* due to lack of expression in all tissues/organs. The expression data of the bZIP genes of two species were row scale normalized respectively after log2^FPKM+1^ by using TBtools (Chen et al., 2020a), and displayed in the heatmap by iTOL (**Figure 1**; **Table S1**).

## Group A bZIPs regulate abiotic stress responses, plant development, and flowering

We further classified 13 *Arabidopsis* and 31 soybean members in group A, characterized as having conserved motifs containing phosphorylation sites, into four subgroups (Jakoby et al., 2002; Dröge-Laser et al., 2018). The first subgroup contained AtbZIP15 and AtbZIP35-AtbZIP38, which are involved in abscisic acid (ABA) and stress signaling (Choi et al., 2000; Uno et al., 2000). ABA is a plant hormone that regulates diverse processes including stomatal closure, osmotic stress response, and seed maturation and germination (Yoshida et al., 2015b). The members of this first subgroup contained abscisic acid responsive element (ABRE) binding factors (ABFs) that function at the core of ABA signaling (Banerjee and Roychoudhury, 2017). Under osmotic stress conditions (such as drought and high salinity), accumulating ABA is perceived by Pyrabactin resistance 1

(PYR1)/PYR1-LIKE (PYL)/Regulatory components of ABA receptor (RCAR) receptors that inhibit the phosphatase activities of protein phosphatase type 2Cs (PP2Cs). ABA-PYR1/PYL/RCAR-PP2C complexes activate (Sucrose non-fermenting-1) SNF1-related protein kinase 2s (SnRK2s), then SnRK2s directly phosphorylate ABFs to strongly enhance their transactivation properties by directly binding to ABRE *cis*-elements (Yoshida et al., 2015b). We identified four GmbZIP TFs in this subgroup, of which *GmbZIP14*, *GmbZIP59*, *GmbZIP50*, and *GmbZIP33* were mainly expressed in roots and flowers (**Figure 1**). Because they clustered with AtbZIP35-AtbZIP 38 in the cladistic tree, they likely also participate in ABA and stress signaling. This idea is reinforced by the detailed analysis of GmbZIP14 (name in the reference is listed in **Table 1**), which is localized in the nucleus and is responsive to ABA, drought, high salinity, and low temperature. Overexpressing *GmbZIP14* improves tolerance to high salt, low temperature, and drought in transgenic plants. Furthermore, some ABREs exist in the promoter region of GmbZIP14 targets, such as *ABA insensitive 1* (*ABI1*), *ABI2*, *RD29B*, *RAB18*, *KAT1*, and *KAT2*, whose expression is also affected by ABA, drought, and high salinity (Gao et al., 2011).

**Table 1:** Well studied soybean bZIP transcription factors.

Group ID	Gene name	Gene name in references	Gene ID	Function	Reference
A	GmbZIP14	GmbZIP1	Glyma.02G131700	Salt, drought and low temperature stresses responses	Gao et al. (2011)
	GmbZIP35	AREB3-1	Glyma.04G124200	Seed development	Jo et al. (2020)
	GmbZIP57	AREB3-2	Glyma.06G314400	Seed development	Jo et al. (2020)
	GmbZIP30	GmFDc1	Glyma.04G022100	Flowering: stem growth habit	Yue et al. (2021)
	GmbZIP50	GmFDL06	Glyma.06G040400	Flowering	Takeshima et al. (2019)
	GmbZIP103	GmFDL12	Glyma.12G184432	Flowering	Nan et al. (2014): Takeshima et al. (2019)
	GmbZIP146	GmFDL19	Glyma.19G122800	Flowering; salt and drought stresses responses	Nan et al. (2014): Takeshima et al. (2019): Li et al. (2017)
C	GmbZIP27	GmbZIP105	Glyma.03G247100	Pathogen response	Alves et al. (2015)
	GmbZIP53	GmbZIP62	Glyma.06G079800	ABA signaling; salt and low temperature stresses responses; pathogen response	Liao et al., 2008; Alves et al. (2015)
	GmbZIP76	G/HBF-1, SBZ1	Glyma.10G162100	Pathogen response	Dröge-Laser et al. (1997); Yoshida et al. (2008)
	GmbZIP159	GmbZIP159	Glyma.20G224500	Seed development	Hu et al. (2022)
D	GmbZIP92	GmbZIP67	Glyma.11G183700	Seed development	Jo et al. (2020)
	GmbZIP104	GmTGA17	Glyma.12G184500	Salt and drought stresses responses	Li et al. (2019)
E	GmbZIP45	GmbZIPE2	Glyma.05G168100	Pathogen response	Alves et al. (2015)
G	GmbZIP28	GmbZIP78	Glyma.03G255000	ABA signaling; salt and low temperature stresses responses	Liao et al., 2008
	GmbZIP82	SGBF-1	Glyma.11G065000	Cold stress response	Kim et al. (2001)
H	GmbZIP142	STF1	Glyma.18G117100	Photomorphogenic; shade avoidance syndrome; lightsignal and nodulation	Shin et al. (2016): Lyu et al. (2021): Ji et al. (2021)
	GmbZIP69	STF2	Glyma.08G302500	Shade avoidance syndrome; light signal and nodulation	Lyu et al. (2021): Ji et al. (2021)
	GmbZIP131	STF3	Glyma.16G092700	Lightsignal and nodulation	Wang et al. (2021)
	GmbZIP20	STF4	Glyma.03G081700	Light signal and nodulation	Wang et al. (2021)
K	GmbZIP15	GmbZIP15	Glyma.02G161100	Salt and drought stresses responses	Zhang et al. (2020)
M	GmbZIP115	GmbZIPE1	Glyma.13G292800	Pathogen response	Alves et al. (2015)
S	GmbZIP8	GmbZIP60	Glyma.02G012700	ABA signaling; salt stress response	Xu et al. (2015)
	GmbZIP32	GmbZIP44	Glyma.04G029600	ABA signaling; salt and low temperature stresses responses	Liao et al., 2008
	GmbZIP51	GmbZIP2	Glyma.06G048500	Salt and drought stresses responses	Yang et al. (2020)
	GmbZIP61	GmbZIP110	Glyma.08G115300	Salt stress response; root growth	Xu et al. (2016): Manavalan et al. (2015)
	GmbZIP97	GmbZIP97	Glyma.12G040600	Seed development	Hu et al. (2022)
	GmbZIP152	GmbZIP152	Glyma.19G216200	Pathogen response; salt, drought, and heavy metal stress responses	Chai et al. (2022)

The uppercase letters are group IDs.

**Figure 2:**
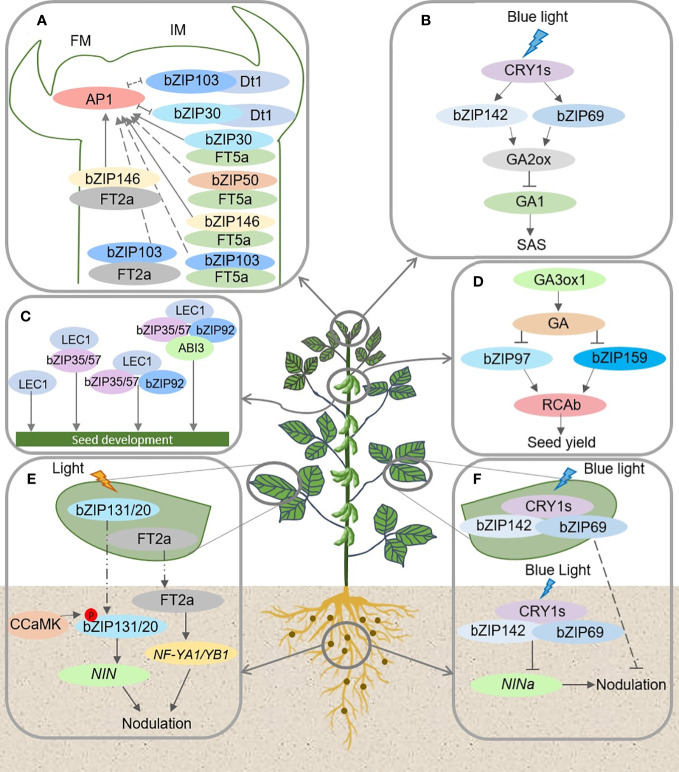
Regulation mechanisms of soybean bZIP TFs involved in selected signaling pathways. **(A)** The soybean bZIP TFs FDc1/bZIP30, FDL06/bZIP50, FDL12/bZIP103, and FDL19/bZIP146 function together with Dt1, FT2a, and/or FT5a in the regulation of flowering and stem growth habit. **(B)** STF1/bZIP142 and STF2/bZIP69, whose abundances are increased by light-activated CRY1s, directly upregulate the expression of genes encoding gibberellin 2 oxidases to deactivate gibberellin 1 and control the blue light-induced shade avoidance syndrome. **(C)** The soybean bZIP TFs ABA-responsive element binding protein 3-1 (AREB3-1/bZIP35), AREB3-2/bZIP57, and bZIP92 regulate distinct seed development processes by acting with LEC1. **(D)** bZIP97 and bZIP159 are involved in gibberellin biosynthesis, which is associated with soybean seed yield. **(E)** STF3/bZIP131-STF4/bZIP20-FT module integrates the light-induced shoot signal and the rhizobium-activated root signal, which coordinately promote nodule formation. **(F)** CRY1s interact with and activate STF1/bZIP142 and STF2/bZIP69 transcription in shoots and roots, which repress *NINa* expression, thereby inhibiting nodulation. FM: Floral meristem, IM: Inflorescence meristem, SAS: Shade avoidance syndrome. The solid arrows and the solid blunt ended arrows represent the stimulation and inhibition effect on downstream gene or substances, respectively. The dashed lines represent the stimulation remains to be further confirmed. ^–..–^ represent movement of proteins.

The second subgroup contained ABI5/DPBF1/AtbZIP39, which has been extensively characterized in *Arabidopsis* and functions in ABA-dependent seed maturation and germination (Lopez-Molina et al., 2001; Skubacz et al., 2016). We classified 16 GmbZIP TFs into this subgroup, most of which were highly expressed in seeds (**Figure 1**), suggesting their potential functions in seed development and germination. In soybean, seed weight is one of the most important yield determinants (Smith and Camper, 1970). The GmbZIP TFs ABA-responsive element binding protein 3-1 (AREB3-1/GmbZIP35), AREB3-2/GmbZIP57, and GmbZIP92 (belonging to Group D) regulate distinct seed development processes by acting with LEAFY COTYLEDON1 (LEC1), a central TF of seed development that controls embryo morphogenesis, photosynthesis, and seed maturation (Jo et al., 2019). LEC1 alone and the LEC1-AREB3 module primarily regulate genes involved in embryo morphogenesis. LEC1-AREB3, LEC1-AREB3-GmbZIP92, and LEC1-AREB3-GmbZIP92-ABI3 modules regulate genes involved in photosynthesis. The LEC1-AREB3-GmbZIP92-ABI3 module also regulates seed maturation genes (Jo et al., 2020) (**Figure 2C**). Soybean *FD-like 19* (*GmFDL19*, *GmbZIP146*) also classified in this subgroup, is highly induced by ABA, polyethylene glycol (PEG 6000), and high salinity. Overexpressing *GmFDL19* in soybean enhances drought and salt tolerance at the seedling stage. Furthermore, *GmFDL19/GmbZIP146* overexpression reduces Na^+^ ion accumulation and up-regulates the expression of several ABA- and stress-responsive genes (Li et al., 2017).

The third subgroup, containing FD (AtbZIP14) and FD PARALOG (FDP, AtbZIP27), is involved in control of the *Arabidopsis* floral transition (Abe et al., 2005). The AtbZIP TF FD promotes flowering with the florigen FLOWERING LOCUS T (FT) as a florigen activation complex (Abe et al., 2005; Wigge et al., 2005). TERMINAL FLOWER 1 (TFL1) competes with FT for FD binding and represses the transcription of floral meristem identity genes, such as *LEAFY* (*LFY*) and *APETALA 1* (*AP1*) (Gustafson-Brown et al., 1994; Hanano and Goto, 2011). GmTFL1b (Dt1), the soybean ortholog of *Arabidopsis* TFL1, controls stem growth habit (Liu et al., 2010; Tian et al., 2010) and flowering time (Yue et al., 2021), which strongly influence soybean grain yield (Bernard, 1972; Heatherly and Smith, 2004; Cao et al., 2016). Soybean contains five FD and FDP homologs, which all belong to this subgroup (Sussmilch et al., 2015) (**Figure 1**). Dt1 interacts with GmFDc1/GmbZIP30 and binds to ACGT *cis*-elements in the promoter region of *GmAP1a* to repress its activity during plant height and flowering time regulation in soybean (Chen et al., 2020b; Yue et al., 2021). Overexpressing *GmFDc1*/*GmbZIP30* leads to early flowering and fewer nodes, suggesting that GmFDc1/GmbZIP30 functions as a floral transition activator (Yue et al., 2021). GmFT5a interferes with Dt1 for GmFDc1 binding and enhances the positive effect of GmFDc1/GmbZIP30 on *GmAP1* expression (Yue et al., 2021). GmFT2a and GmFT5a both induce flowering; however, GmFT5a plays an additional role in termination of shoot apical meristem growth shortly after floral induction (Takeshima et al., 2019). GmFT5a, but not GmFT2a, competes with Dt1 for GmFDc1/GmbZIP30 binding to more rapidly terminate stem growth (Takeshima et al., 2019; Yue et al., 2021) (**Figure 2A**). The functions of the other four FD and FDP homologs are not known, and can only be inferred from their gene expression patterns: *GmbZIP9* is mainly expressed in shoot apices and seeds, *GmbZIP48*, *GmbZIP30*, *GmbZIP4* and *GmbZIP161* in shoot apices and stems. These distinct expression patterns indicate their functional differentiation during soybean selection and domestication. Furthermore, three other group A members are also involved in floral transition: GmFDL06/GmbZIP50, which interacts with GmFT5a; GmFDL12/GmbZIP103, which functions together with Dt1, GmFT2a, and GmFT5a; and GmFDL19/GmbZIP146, which associates with GmFT2a and GmFT5a (Nan et al., 2014; Takeshima et al., 2019). *GmFDL19*/*GmbZIP146* overexpression in soybean causes early flowering, which may be mediated by upregulation of floral identity genes, such as *Suppressor of overexpression of CO 1* (*SOC1*s), *LFY*s, and *AP1*s, the possible direct targets of GmFDL19/GmbZIP146 (Nan et al., 2014) (**Figure 2A**).

Group A *Arabidopsis* bZIPs could be divided into four subgroups; three of them have specific functions: ABA and stress signaling, seed maturation and germination, and flowering time and stem growth. Although only a few Group A soybean members are well studied, their functions are largely consistent with those of *Arabidopsis* members. These studies provide directions for studying other members in each subgroup.

## Group B and K bZIPs function in endoplasmic reticulum stress pathways

Two bZIPs (AtbZIP17 and AtbZIP28) in group B and the only member in group K (AtbZIP60) function in two endoplasmic reticulum (ER) stress pathways (Iwata and Koizumi, 2005; Howell, 2013). ER stress occurs under adverse environmental conditions, and the ER stress response is implicated in seed development and pathogen response (Vitale and Ceriotti, 2004). AtbZIP17 and AtbZIP28 regulate the unfolded protein response pathway, and AtbZIP60 is involved in a plant-specific ER stress response signal transduction pathway (Iwata and Koizumi, 2005; Howell, 2013). There are two soybean members in group B, GmbZIP21 and GmbZIP147, whose genes are highly expressed in seeds. Their expression patterns provide clues about their analogous functions in the ER stress response; however, their functions are not clearly understood. The only soybean member in group K (GmbZIP15) is involved in the abiotic stress response, different from its *Arabidopsis* homolog AtbZIP60. *GmbZIP15* transcription is suppressed under salt- and drought-stress conditions. Overexpressing *GmbZIP15* in soybean results in hypersensitivity to abiotic stress compared with wild-type plants, which is associated with lower expression of stress-responsive genes, defective stomatal aperture regulation, and reduced antioxidant enzyme activities (Zhang et al., 2020). Considering the crucial functions of AtbZIP60 in the ER stress response, the functions of GmbZIP15 deserve more attention.

## Group C and S bZIPs regulate stress responses and plant development

In *Arabidopsis*, four group C members preferentially heterodimerize with five group S1 members, which is defined as the ‘C/S1 bZIP network’ (Jakoby et al., 2002; Weltmeier et al., 2006). The C/S1 bZIP network is involved in metabolic readjustment during low-energy signaling, downstream of SnRK1 (Baena-González et al., 2007). SnRK1-C/S1 signaling is involved in pathogen defense, which is an energy-consuming process requiring metabolic readjustment. Likewise, C/S1 bZIPs, such as AtbZIP10 in *Arabidopsis* and AtbZIP63 orthologs in several plant species, are implicated in pathogen defense (Dröge-Laser et al., 1997; Kuhlmann et al., 2003; Kaminaka et al., 2006; Shen et al., 2016). We detected 8 and 28 soybean members in groups C and S, respectively, and most of them are expressed in roots and stems, similar to their *Arabidopsis* counterparts (**Figure 1**). Dröge-Laser et al. (1997) isolated a group C member, G/HBF-1/GmbZIP76, which activates pathogen defense by binding to the promoters of *Chalcone Synthase 15* (*CHS15*), *CHS7*, and *CHS1*, which belong to the chalcone synthase (CHS) family that catalyzes the first and key regulatory step of flavonoid biosynthesis, the well-characterized defense substances (Dixon et al., 1983; Hahlbrock and Scheel, 1989; Wingender et al., 1990; Yoshida et al., 2008). *In-vitro* phosphorylation of G/HBF-1/GmbZIP76 enhances its binding to the *CHS15* promoter. A cytosolic serine kinase that is stimulated by an avirulent strain of the soybean pathogen *Pseudomonas syringae* pv. *glycinea* was identified using recombinant G/HBF-1/GmbZIP76 as a substrate. Stimulation of G/HBF-1/GmbZIP76 kinase activity and G/HBF-1/GmbZIP76 phosphorylation are terminal events in a signal pathway to activate early transcription-dependent plant defense responses (Dröge-Laser et al., 1997). Genes encoding the Group C proteins GmbZIP53 and GmbZIP27 are differentially expressed in the resistant soybean cultivar PI561356 during Asian soybean rust (ASR) infection, which is caused by an obligate biotrophic pathogenic fungus *Phakopsora pachyrhizi* and results in yield losses of up to 80%, indicating their important roles in the response to ASR infection (Patil et al., 1997; Alves et al., 2015).

Moreover, some *Arabidopsis* C and S1 members are also involved in abiotic stress responses, such as AtbZIP1 and its partners AtbZIP53, AtbZIP10, and AtbZIP25 (Sun et al., 2012; Hartmann et al., 2015). In soybean, GmbZIP61, GmbZIP51, GmbZIP8, and GmbZIP32 in groups C and S positively regulate drought and salt stress responses; and GmbZIP53 positively regulates drought, salt, and low-temperature stress responses; GmbZIP152 positively regulate drought, salt, heavy metal, and *Sclerotinia sclerotiorum* stress responses (Liao et al., 2008; Xu et al., 2015; Xu et al., 2016; Yang et al., 2020; Chai et al., 2022). (*GmbZIP61* overexpression in *Arabidopsis* improved salt tolerance by elevating the survival rate, rosette diameter, relative electrolyte leakage, and proline content after a 200 mM NaCl treatment. GmbZIP61 binds to the ACGT motif in promoters and influences the expression of many stress-related genes as well as the accumulation of proline, Na^+^, and K^+^ (Xu et al., 2016). *GmbZIP51* is induced by multiple abiotic stresses. *GmbZIP51* overexpression in *Arabidopsis* and soybean hairy roots improves tolerance to drought and salt stresses and enhances the expression of the stress-responsive genes *GmMYB48*, *GmWD40*, *Dehydrins 15* (*GmDHN15*), *Glutathione S-transferase 1* (*GmGST1*), and *Late Embryogenesis Abundant* (*GmLEA*) (Yang et al., 2020). GmbZIP8 is implicated in abiotic stress responses. *GmbZIP8* is induced by ABA and salt stress. Promoter analysis indicated that the *GmbZIP8* promoter contains *cis*-acting elements involved in defense and stress responses, such as the ABREs involved in the ABA response and the MYB binding site involved in the drought response. *Arabidopsis* plants heterologously expressing the *GmbZIP8* promoter indicated that *GmbZIP8* is strongly induced by ABA and weakly induced by salt (Xu et al., 2015). Liao et al. (2008) reported that *GmbZIP32* and *GmbZIP53* are differentially regulated by various treatments. *GmbZIP32* is induced by drought, flooding, and salt stress, but moderately induced by ABA treatment; *GmbZIP53* is slightly induced by drought and salt treatments. Transgenic *Arabidopsis* plants overexpressing *GmbZIP32* or *GmbZIP53* had reduced ABA sensitivity, but enhanced salt and low-temperature stress tolerance. *GmbZIP152* is significantly induced by salt, drought, heavy metal, and *S. sclerotiorum* stresses in soybean. Overexpression of *GmbZIP152* in *Arabidopsis* enhances the resistance to the abiotic and *S. sclerotiorum* stresses. ABA-, JA-, ETH-, and SA-induced biotic- and abiotic-related genes, such as *Early Response to Dehydration 1* (*GmERD1*) and *Pathogenesis-related 2* (*GmPR2*), might be the direct targets of GmbZIP152 (Chai et al., 2022).

The C/S1 bZIP network is also implicated in seed development. The Group C members in *Arabidopsis* (AtbZIP10 and AtbZIP25) control seed storage protein biosynthesis; AtbZIP53 activates seed maturation by interacting with AtbZIP10 and AtbZIP25 (Lara et al., 2003; Alonso et al., 2009). GmbZIP97 (Group S) and GmbZIP159 (Group C) are involved in gibberellin biosynthesis, which is associated with soybean hundred-seed weight and seed number. Knocking out *Gibberellin 3-oxidase1* (*GmGA3ox1*), which encodes a key gibberellin biosynthesis enzyme, decreases the content of bioactive gibberellins in leaves while enhancing photosynthesis, thereby promoting seed yield by upregulating *GmbZIP97* (Group S) and *GmbZIP159* (Group C), and then GmbZIP97 and GmbZIP159 directly activate ribulose-1,5-bispho-sphate carboxylase-oxygenase (Rubisco) activases (*GmRCAb*). Further, GmRCAb induces the production of more Rubisco to increase photosynthesis and ensures sufficient energy transport from leaves to seeds (Hu et al., 2022) (**Figure 2D**).

Because many genes encoding Group S members are expressed in soybean roots, some of them are implicated in regulating root size and architecture, which are important for yield performance (Price et al., 2002; Manavalan et al., 2009). A major quantitative trait locus on chromosome 8 (the Satt315-I locus) controls tap root length, lateral root number, and shoot length in soybean. Eleven TF genes were identified within the confidence interval of this region, among them, the Group S member *GmbZIP61* is highly expressed in the root pericycle and nodules. Pericycle cells form lateral root primordium, which determine lateral root number (Manavalan et al., 2015). *GmbZIP43*, closely related to *GmbZIP61*, is also highly expressed in roots, which suggests its similar function in regulating root architecture.

*Arabidopsis* Group C members always heterodimerize and work together with Group S1 members; however, this phenomenon has not been reported in soybean. Therefore, soybean C and S members may have similar biochemical properties, but further study at the molecular level is needed.

## Group D members have diverse functions

Characterized by a short zipper domain, a conserved C terminus, and a more variable N terminus, Group D comprises the so-called ‘TGACG motif-binding factors’ (TGAs), which are further divided into five clades in Arabidopsis (Gatz, 2013). AtTGA1/AtbZIP47 and AtTGA4/AtbZIP57 in Clade I participate in root nitrate uptake, nitrate responses, apoplastic defenses, ER stress responses, salicylic acid biosynthesis, and pathogen defense (Miao et al., 1994; Wang and Fobert, 2013; Alvarez et al., 2014; Sun et al., 2018). The *Arabidopsis* Clade II factors AtTGA2/AtbZIP20, AtTGA5/AtbZIP26, and AtTGA6/AtbZIP45 play crucial roles in systemic acquired resistance and detoxification processes (Xiang et al., 1997; Müller and Berger, 2008; Fu and Dong, 2013). AtTGA3/AtbZIP22 in Clade III is involved in basal pathogen resistance and in mediating phytohormonal cross-talk between salicylic acid and cytokinin (Choi et al., 2010). *Arabidopsis* Clade IV members AtTGA9/AtbZIP21 and AtTGA10/AtbZIP65 regulate anther development (Murmu et al., 2010). The *Arabidopsis* Clade V member AtTGA8/PERIANTHIA (AtPAN/AtbZIP46) controls the formation of floral organ primordia (Chuang et al., 1999; Maier et al., 2011). The soybean members in each clade were expressed in distinct tissues (**Figure 1**), which suggests their differential roles in soybean. *TGACG motif-binding factor 17* (*GmTGA17*, *GmbZIP104*), encoding a Clade IV protein, is strongly induced by drought and salt stress. Heterologous overexpression of *GmTGA17* in *Arabidopsis* and homologous overexpression in soybean hairy roots enhanced drought and salt tolerance (Li et al., 2019). *GmTGA17/GmbZIP104* is highly expressed in roots, stems, and flowers (**Figure 1**), which indicated its potential function in another development, similar to AtTGA9/AtbZIP21 and AtTGA10/AtbZIP65.

## Group E and M bZIPs regulate pollen wall formation and biotic stress responses

We identified six *Arabidopsis* and four soybean bZIP TFs in Group E. Research on *Arabidopsis* Group E bZIP TFs is limited. Only one study reported that one member from Group E, AtbZIP34, has an essential role in pollen wall formation, and the *atbzip34* mutants show pollen morphology and pollen germination defects. AtbZIP34 is involved in lipid metabolism, cellular transport, and intine biosynthesis by regulating the putative downstream gene *MYB97* (Gibalová et al., 2009). The soybean bZIP TFs GmbZIP106 and GmbZIP114 are closely related to AtbZIP34. *GmbZIP106* and *GmbZIP114* are highly expressed in flowers, suggesting their involvement in floral organ development (**Figure 1**).

Two other soybean bZIP TF genes, *GmbZIPE1*/*GmbZIP115* (Group M) and *GmbZIPE2*/*GmbZIP45* (Group E), are differentially expressed during ASR infection in the resistant soybean cultivar PI561356, indicating that their proteins participate in the response to ASR infection (Alves et al., 2015), but their exact functions and regulation mechanisms need further study. All soybean Group M members are highly expressed in seeds, indicating that they have similar functions (**Figure 1**).

## Group F bZIPs regulate zinc deficiency and salt stress responses

*Arabidopsis* Group F members AtbZIP19 and AtbZIP23 are essential for adaptation to zinc deficiency in *Arabidopsis* roots (Assuncão et al., 2010). AtbZIP24 is an important regulator of the salt stress response; transcriptional repression of *AtbZIP24* improves salt tolerance in *Arabidopsis* (Yang et al., 2009). GmbZIP84 in this group, whose gene is also highly expressed in roots, may have similar functions as its *Arabidopsis* homologs (AtbZIP19, AtbZIP23, and AtbZIP24).

## Group G bZIPs regulate abiotic stress responses

Group G proteins are characterized by a proline-rich N-terminal activation domain (Jakoby et al., 2002). G-BOX-BINDING FACTOR1 (AtGBF1), a well-known *Arabidopsis* Group G bZIP TF, regulates blue-light-dependent hypocotyl expansion, lateral root development, natural senescence, and salicylic acid-dependent pathogen defense (Weisshaar et al., 1991; Schindler et al., 1992; Smykowski et al., 2010; Maurya et al., 2015; Giri et al., 2017). We identified 14 soybean bZIPs in Group G, and Soybean G-box binding factor 1 (SGBF-1, GmbZIP82), GmbZIP6, GmbZIP10, and GmbZIP133 are homologs of *Arabidopsis* GBF1, and their genes are mainly expressed in flowers and leaves (**Figure 1**). SGBF-1/GmbZIP82 has been extensively studied and participates in abiotic stress responses. SGBF-1/GmbZIP82 binds directly to ABREs in cold-regulated gene promoters. SGBF-1/GmbZIP82 interacts with the C2H2-type zinc finger protein SCOF-1 to up-regulate *COLD-REGULATED* (*AtCOR*) expression and enhance cold tolerance in transgenic *Arabidopsis* (Kim et al., 2001). Another well-studied soybean Group G member is *GmbZIP28*, which is slightly induced by NaCl treatment. Transgenic *Arabidopsis* plants overexpressing *GmbZIP28*showed reduced ABA sensitivity, but increased salt and low-temperature tolerance. GmbZIP28 binds to GLM (GTGAGTCAT), ABRE (CCACGTGG), and PB-like (TGAAAA) *cis*-elements and may function in ABA signaling by upregulating *ABI1* and *ABI2*, and has roles in stress tolerance by regulating various stress-responsive genes (Liao et al., 2008). Also in Group G, GmbZIP154, GmbZIP58, and GmbZIP130 are homologs of AtGBF2 and AtGBF3; and GmbZIP72, GmbZIP1, GmbZIP68, and GmbZIP132 are homologs of AtbZIP16 and AtbZIP68, but their functions are not clearly understood.

## Group H bZIPs regulate environmental signaling and carbon-nitrogen balance

Group H contains only two *Arabidopsis* members, ELONGATED HYPOCOTYL5 (AtHY5, AtbZIP56) and HY5 HOMOLOG (AtHYH, AtbZIP64). AtHY5/AtbZIP56 promote photomorphogenesis downstream of phytochromes, cryptochromes, and UV-B photoreceptors, and regulates cell elongation, cell proliferation, chloroplast development, lateral root development, pigment accumulation, and nutrient assimilation (Gangappa and Botto, 2016). AtHYH/AtbZIP64 forms heterodimers with AtHY5/AtbZIP56 and enhances transcriptional activation; AtHYH/AtbZIP64 acts redundantly with AtHY5/AtbZIP56 to regulate hypocotyl growth, lateral root growth, pigment accumulation, and the expression of light-inducible genes (Holm et al., 2002; Gangappa and Botto, 2016). Four soybean bZIP TFs belong to this group. Soybean TGACG-motif-binding factor 1 (STF1, GmbZIP142) and STF2/GmbZIP69, mainly expressed in leaves, are homologs of AtHY5. STF3/GmbZIP131 and STF4/GmbZIP20, mainly expressed in leaves, are homologs of AtHYH (**Figure 1**).

STF1/GmbZIP142 plays a positive role in photomorphogenesis and phytohormone signaling (Song et al., 2008). The C terminus of STF1/GmbZIP142 complemented the *Athy5 Arabidopsis* mutant phenotype for hypocotyl length, root gravitropic response, and chlorophyll and anthocyanin content, indicating their analogous roles in *Arabidopsis* and soybean. STF1/GmbZIP142 interacts with three B-box zinc finger proteins STO homolog (GmSTH), and GmSTH2 and with Constitutively Photomorphogenic 1 (GmCOP1), which play important roles in light-dependent development and gene expression. The regulatory mechanisms that involve GmCOP1, STF1/GmbZIP142, and the B-box factors in soybean may be similar to those in *Arabidopsis*, including STF1/GmbZIP142, GmSTO, and GmSTH degradation in the dark *via* the GmCOP1-mediated ubiquitination pathway (Shin et al., 2016). In addition, both AtHY5/AtbZIP56 and STF1/GmbZIP142 have strong binding affinity to ACGT-containing elements, suggesting that these two proteins have similar functions and may regulate similar downstream genes (Song et al., 2008).

AtHY5/AtbZIP56 also participates in the shade avoidance response; *AtHY5*/*AtbZIP56* and *AtHYH*/*AtbZIP64* are induced by low red/far-red light ratios (Ciolfi et al., 2013). Soybean displays the classic shade avoidance syndrome, including exaggerated stem elongation, which leads to lodging and yield reduction under dense planting conditions. Two AtHY5/AtbZIP56 homologs in soybean, STF1/*GmbZIP142* and STF2/GmbZIP69, whose abundances are increased by light-activated Cryptochrome Circadian Regulator 1 (GmCRY1s), directly upregulate the expression of genes encoding gibberellin 2 oxidases to deactivate gibberellin 1 and repress stem elongation and control the blue light-induced shade avoidance syndrome. *GmCRY1b* overexpression lines are promising lodging-resistant options for dense planting and intercropping conditions (Lyu et al., 2021) (**Figure 2B**).

In *Arabidopsis*, another important role of AtHY5/AtbZIP56 is to adjust the carbon-nitrogen balance. In leaves, AtHY5/AtbZIP56 activates the transcription of SWEET-facilitator genes to support sucrose export to roots (Chen et al., 2016). AtHY5/AtbZIP56 moves from shoot to root to activate its own expression to promote nitrate uptake by activating *NITRATE TRANSPORTER2.1* (*AtNRT2.1*) expression (Chen et al., 2016). In roots, AtHY5/AtbZIP56 is involved in nitrogen signaling pathways by positively regulating *NITRATE REDUCTASE2* (*AtNIA2*) and *NITRITE REDUCTASE1* (*AtNIR1*) expression, and negatively regulating *AtNRT1.1* and *AMMONIUM TRANSPORTER1;2* (*AtAMT1;2*) expression (Yanagisawa, 2014; Huang et al., 2015). Different from *Arabidopsis*, legumes evolved a symbiotic relationship with rhizobia, who fix atmospheric nitrogen and provide nitrogen nutrients to their host plant, and the soybean AtHY5 homologs STF1/GmbZIP142 and STF2/GmbZIP69 are also involved in light-mediated symbiotic root nodulation (Wang et al., 2021). Specifically, the blue light receptor GmCRY1-STF1/GmbZIP142-STF2/GmbZIP69 module plays a pivotal role in integrating darkness/blue light and nodulation signals. Soybean perceives blue light by GmCRY1s, which activates *STF1*/*GmbZIP142* and *STF2*/*GmbZIP69* transcription in shoots and roots. Root GmCRY1s interact with and elevate the levels of STF1/GmbZIP142 and STF2/GmbZIP69, which repress *Nodule Inception a* (*GmNINa*) expression, thereby inhibiting nodulation (Ji et al., 2021) (**Figure 2F**). Wang et al. (2021) demonstrated that light-induced STF3/GmbZIP131, STF4/GmbZIP20, and GmFTs interdependently induce nodule organogenesis from shoots to roots. The rhizobium-activated calcium- and calmodulin-dependent protein kinase (CCaMK) phosphorylates STF3/GmbZIP131, triggering STF3/GmbZIP131-FT2a complex formation, which directly activates *GmNIN* and *Nuclear factor Y* (*GmNF-YA1* and *GmNF-YB1*) expression. The GmCCaMK-STF3/GmbZIP131-STF4/GmbZIP20-FT module integrates the light-induced shoot signal and the rhizobium-activated root signal, which coordinately promote nodule formation (**Figure 2E**).

## Group I bZIPs regulate plant development

We identified 12 *Arabidopsis* and 17 soybean members in Group I, who share a characteristic lysine residue in the basic domain that replaces the highly conserved arginine (Jakoby et al., 2002). The best-studied *Arabidopsis* member in group I is VIRE2-INTERACTING PROTEIN 1 (AtVIP1, AtbZIP51), which is involved in the *Agrobacterium tumefaciens* response, pathogen response, abiotic stress response, cell proliferation, and plant development (Van Leene et al., 2016). The most closely related soybean bZIP TFs are GmbZIP11, GmbZIP134, and GmbZIP78. GmbZIP11 and *GmbZIP134* are highly expressed in leaves and stems; *GmbZIP78* is highly expressed in seeds (**Figure 1**).

Group I members regulate plant development. AtbZIP29 regulates the cell number in leaves and root meristems by controlling cell wall organization, and DRINK ME (AtDKM, AtbZIP30) affects meristematic tissues and gynoecium development (Lozano-Sotomayor et al., 2016; Van Leene et al., 2016). Their homologous genes in soybean, *GmbZIP85*, *GmbZIP139*, and *GmbZIP31*, are highly expressed in flowers, which implies their similar roles as AtDKM, whereas *GmbZIP124* and *GmbZIP96* are highly expressed in roots and stems, which suggests similar functions as those of AtbZIP29. Another Group I member, AtbZIP18, controls pollen development by interacting with AtbZIP34, AtbZIP52, and AtbZIP61, and works redundantly with AtbZIP34 (Gibalová et al., 2017). AtbZIP18, AtbZIP52, AtbZIP34, and AtbZIP61 have one to four homologs in soybean; their unknown redundancy may make it difficult to study their functions. In Group I, AtbZIP59 interacts with lateral organ boundaries domain (LBD) TFs to regulate auxin-induced callus formation, which is required for plant regeneration (Xu et al., 2018). *GmbZIP87*, *GmbZIP99*, *GmbZIP118*, and *GmbZIP125* are mainly expressed in roots and stems, but their exact functions are unknown.

## Conclusions and perspectives

Plant bZIP TFs regulate a variety of biological processes. The functions of some soybean bZIP TFs have been extensively studied (**Table 1**). Most soybean members in Groups B, K, D, E, M, and G regulate abiotic stress responses, which differs from the various functions of *Arabidopsis* members in these groups, such as ER stress response, root nitrate uptake, nitrate responses, apoplastic defenses, pathogen defense, floral organ development, pollen development, lateral root development, and natural senescence. However, the functions of soybean members in Groups A, C, S, and H are highly consistent with those of *Arabidopsis* members in these groups. Our knowledge of soybean members in Groups B, K, D, E, M, and G is limiteds, and reports on the functions of Group F, I, J, and N soybean members are even less clear. Therefore, detailed investigations of all soybean bZIP TF functions and molecular mechanisms are needed. The cladistic, transcriptional, and functional information we provided in each group will be useful for future studies.

Soybean is an important protein and oil crop. Soybean yield is determined by multiple traits, including flowering time, node number, internode length, effective branching number, pod number per plant, seed number per plant, and hundred-seed weight (Pedersen and Lauer, 2004; Lu et al., 2017; Lu et al., 2020). Soybean bZIP TFs regulate many important yield traits. For example, GmFDc1, GmFDL12, GmFDL19, and GmFDL06 (Group A) are transcription cofactors of Dt1, GmFT2a, and/or GmFT5a, which determine stem growth habit and/or flowering time of soybean (Nan et al., 2014; Takeshima et al., 2019; Li et al., 2021; Yue et al., 2021). The detailed functions and regulatory mechanisms of these Group A TFs remain unclear, but they appear to have enormous contributions to soybean yield. STF1 and STF2 (Group H) are involved in the shade avoidance syndrome, which corresponds to internode length of soybean main stems (Lyu et al., 2021). AREB3 (Group A) and GmbZIP92 (Group D) regulate seed development by acting with LEC1 (Jo et al., 2020). GmbZIP97 (Group S) and GmbZIP159 (Group C) are involved in the gibberellin-mediated seed development pathway (Hu et al., 2022). These bZIP TFs are optimal gene resources for soybean breeding. Crop yield is reduced when plants are exposed to extreme environmental conditions such as high salt, drought, cold, and heat, as well as to biotic stresses such as insects and pathogen invasion. Plants exhibit numerous adaptive and protective responses to various abiotic and biotic stimuli. Most of the soybean bZIP TFs are considered abiotic stress regulators; about 75.6% of soybean bZIP genes display transcriptional changes after abiotic stress treatment (Zhang et al., 2018). Among the well-studied soybean bZIP TFs, 11 regulate abiotic stress responses, and 5 are involved in biotic stress responses (**Table 1**). Most of the environmental-stress-related *Arabidopsis* bZIP TFs are distributed in Groups A, C, S, B, K, D, G, and F. Although the functional diversity and molecular mechanisms of these soybean members need further study, preliminary data provide a valuable basis for future study.

Biological nitrogen fixation is an alternative to nitrogen fertilizer; the ability of legumes to form a symbiosis with nitrogen-fixing rhizobia provides a distinct advantage (Ferguson et al., 2010). STF1, STF2, STF3, and STF4 (Group H) are orthologs of AtHY5 and AtHYH. STF1 and STF2 suppress soybean nodulation, while STF3 and STF4 play positive roles in light-induced nodulation responses in soybean (Ji et al., 2021; Wang et al., 2021). Further studies are needed to determine why these proteins regulate nodulation in different ways, and different strategies should be used to utilize these gene resources in soybean breeding.

The functions of some soybean bZIP TFs have been analyzed *via* physiological experiments and genetic engineering; however, the biochemical properties and regulation mechanisms of many members remain unclear. Because of the duplication of the soybean genome, soybean contains two to ten bZIP TF homologs, which makes it difficult to analyze their homodimers/heterodimers, binding sites, and knockout phenotypes. Advances in genomics and molecular biology have facilitated cloning of homologs and screening of TF binding sites, and clustered regularly interspaced short palindromic repeats (CRISPR)/CRISPR-associated protein 9 (Cas9) gene editing technologies have accelerated the generation of single and multiple mutants for studying genes and their functions, which will also facilitate the study of soybean bZIP TFs.

## Author contributions

XL, LZ, and FK conceptualized the idea, LY and XP wrote the initial manuscript draft. All authors contributed to the article and approved the submitted version.

## References

[B1] Abe M. Kobayashi Y. Yamamoto S. Ichinoki H. Notaguchi M. Goto K. (2005). FD, a bZIP protein mediating signals from the floral pathway integrator FT at the shoot apex. Science. 309, 1052–1057. doi: 10.1126/science.1115983 16099979

[B2] Albani D. Hammond-Kosack M. C. Smith C. Conlan S. Colot V. Holdsworth M.. (1997). The wheat transcriptional activator SPA: A seed-specific bZIP protein that recognizes the GCN4-like motif in the bifactorial endosperm box of prolamin genes. Plant Cell. 9, 171–184. doi: 10.1105/tpc.9.2.171 9061949 PMC156909

[B3] Alonso R. Oñate-Sánchez L. Weltmeier F. Ehlert A. Diaz I. Dietrich K.. (2009). A pivotal role of the basic leucine zipper transcription factor bZIP53 in the regulation of arabidopsis seed maturation gene expression based on heterodimerization and protein complex formation. Plant Cell. 21, 1747–1761. doi: 10.1105/tpc.108.062968 19531597 PMC2714925

[B4] Alvarez J. M. Riveras E. Vidal E. A. Gras D. E. Contreras-López O. Tamayo K. P.. (2014). Systems approach identifies TGA1 and TGA4 transcription factors as important regulatory components of the nitrate response of arabidopsis thaliana roots. Plant J. 80, 1–13. doi: 10.1111/tpj.12618 25039575

[B5] Alves M. S. Soares Z. G. Vidigal P. M. P. Barros E. G. Poddanosqui A. M. P. Aoyagi L. N. (2015). Differential expression of four soybean bZIP genes during Phakopsora pachyrhizi infection. Funct. Integr. Genomics 15, 685–696. 26013145 10.1007/s10142-015-0445-0

[B6] Assuncão A. G. Herrero E. Lin Y. F. Huettel B. Talukdar S. Smaczniak C.. (2010). Arabidopsis thaliana transcription factors bZIP19 and bZIP23 regulate the adaptation to zinc deficiency. Proc. Natl. Acad. Sci. U.S.A. 107 (22), 10296–10301. doi: 10.1073/pnas.1004788107 20479230 PMC2890486

[B7] Baena-González E. Rolland F. Thevelein J. M. Sheen J. (2007). A central integrator of transcription networks in plant stress and energy signalling. Nature 448, 938–942. doi: 10.1038/nature06069 17671505

[B8] Banerjee A. Roychoudhury A. (2017). Abscisic-acid-dependent basic leucine zipper (bZIP) transcription factors in plant abiotic stress. Protoplasma 254, 3–16. doi: 10.1007/s00709-015-0920-4 26669319

[B9] Bernard R. L. (1972). Two genes affecting stem termination in soybeans. Crop Sci. 12, 235–239. doi: 10.2135/cropsci1972.0011183X001200020028x

[B10] Cao D. Takeshima R. Zhao C. Liu B. Jun A. Kong F. (2016). Molecular mechanisms of flowering under long days and stem growth habit in soybean. J. Exp. Bot. 8, 1873–1884. doi: 10.1093/jxb/erw394 28338712

[B11] Chai M. Fan R. Huang Y. Jiang X. Wai M. H. Yang Q.. (2022). GmbZIP152, a soybean bZIP transcription factor, confers multiple biotic and abiotic stress responses in plant. Int. J. Mol. Sci. 23, 10935. doi: 10.3390/ijms231810935 36142886 PMC9505269

[B12] Chen C. Chen H. Zhang Y. Thomas H. R. Frank M. H. He Y.. (2020a). TBtools: an integrative toolkit developed for interactive analyses of big biological data. Mol. Plant 13 (8), 1194–1202. doi: 10.1016/j.molp.2020.06.009 32585190

[B13] Chen L. Nan H. Kong L. Yue L. Yang H. Zhao Q.. (2020b). Soybean AP1 homologs control flowering time and plant height. j. integr. Plant Biol. 62, 1–12. doi: 10.1111/jipb.12988 32619080

[B14] Chen X. Yao Q. Gao X. Jiang C. Harberd N. P. Fu X. (2016). Shoot-to-root mobile transcription factor HY5 coordinates plant carbon and nitrogen acquisition. Curr. Biol. 26, 640–646. doi: 10.1016/j.cub.2015.12.066 26877080

[B15] Chern M. S. Eiben H. G. Bustos M. M. (1996). The developmentally regulated bZIP factor ROM1 modulates transcription from lectin and storage protein genes in bean embryos. Plant J. 10, 135–148. doi: 10.1046/j.1365-313X.1996.10010135.x 8758983

[B16] Choi H. Hong J. Ha J. Kang J. Kim S. Y. (2000). ABFs, a family of ABA- responsive element binding factors. J. Biol. Chem. 275, 1723–1730. doi: 10.1074/jbc.275.3.1723 10636868

[B17] Choi J. Huh S. U. Kojima M. Sakakibara H. Paek K. H. Hwang I. (2010). The cytokinin-activated transcription factor ARR2 promotes plant immunity *via* TGA3/NPR1-dependent salicylic acid signaling in arabidopsis. Dev. Cell. 19, 284–295. doi: 10.1016/j.devcel.2010.07.011 20708590

[B18] Chuang C. F. Running M. P. Williams R. W. Meyerowitz E. M. (1999). The PERIANTHIA gene encodes a bZIP protein involved in the determination of floral organ number in arabidopsis thaliana. Genes Dev. 13, 334–344. doi: 10.1101/gad.13.3.334 9990857 PMC316427

[B19] Ciolfi A. Sessa G. Sassi M. Possenti M. Salvucci S. Carabelli M.. (2013). Dynamics of the shade-avoidance response in arabidopsis. Plant Physiol. 163, 331–353. doi: 10.1104/pp.113.221549 23893169 PMC3762654

[B20] Cluis C. P. Mouchel C. F. Hardtke C. S. (2004). The arabidopsis transcription factor HY5 integrates light and hormone signaling pathways. Plant J. 38, 332–347. doi: 10.1111/j.1365-313X.2004.02052.x 15078335

[B21] Corrêa L. G. G. Riaño-Pachón D. M. Schrago C. G. Vicentini dos Santos R. Mueller-Roeber B. Vincentz M. (2008). The role of bZIP transcription factors in green plant evolution: adaptive features emerging from four founder genes. PloS One 3 (8), e2944. doi: 10.1371/journal.pone.0002944 18698409 PMC2492810

[B22] Dixon R. A. Dey P. M. Lamb C. J. (1983). Phytoalexins: enzymology and molecular biology. Adv. Enzymol. Relat. Areas. Mol. Biol. 55, 1–136. doi: 10.1002/9780470123010.ch1 6353887

[B23] Dröge-Laser W. Kaiser A. Lindsay W. P. Halkier B. A. Loake G. J. Doerner P.. (1997). Rapid stimulation of a soybean protein-serine kinase that phosphorylates a novel bZIP DNA-binding protein, G/HBF-1, during the induction of early transcription-dependent defenses. EMBO J. 16, 726–738. doi: 10.1093/emboj/16.4.726 9049302 PMC1169674

[B24] Dröge-Laser W. Snoek B. L. Snel B. Weiste C. (2018). The arabidopsis bZIP transcription factor family - an update. Curr. Opin. Plant Biol. 45, 36–49. doi: 10.1016/j.pbi.2018.05.001 29860175

[B25] Ferguson B. J. Indrasumunar A. Hayashi S. Lin M. H. Lin Y. H. Reid D. E. (2010). Molecular analysis of legume nodule development and autoregulation. J. Integr. Plant Biol. 52 (1), 61–76. 20074141 10.1111/j.1744-7909.2010.00899.x

[B26] Fu Z. Q. Dong X. (2013). Systemic acquired resistance: turning local infection into global defense. Annu. Rev. Plant Biol. 64, 839–863. doi: 10.1146/annurev-arplant-042811-105606 23373699

[B27] Fukazawa J. Sakai T. Ishida S. Yamaguchi I. Kamiya Y. Takahashi Y. (2000). REPRESSION OF SHOOT GROWTH, a bZIP transcriptional activator, regulates cell elongation by controlling the level of gibberellins. Plant Cell. 12, 901–915. doi: 10.1105/tpc.12.6.901 10852936 PMC149092

[B28] Gangappa S. N. Botto J. F. (2016). The multifaceted roles of HY5 in plant growth and development. Mol. Plant 9, 1353–1365. doi: 10.1016/j.molp.2016.07.002 27435853

[B29] Gao S. Q. Chen M. Xu Z. S. Zhao C. P. Li L. Xu H.. (2011). The soybean GmbZIP1 transcription factor enhances multiple abiotic stress tolerances in transgenic plants. Plant Mol. Biol. 75, 537–553. doi: 10.1007/s11103-011-9738-4 21331631

[B30] Gatz C. (2013). From pioneers to team players: TGA transcription factors provide a molecular link. Mol. Plant Microbe Interact. 26, 151–159. doi: 10.1094/MPMI-04-12-0078-IA 23013435

[B31] Gibalová A. Reňák D. Matczuk K. Dupl’áková N. Cháb D. Twell D.. (2009). AtbZIP34 is required for arabidopsis pollen wall patterning and the control of several metabolic pathways in developing pollen. Plant Mol. Biol. 70, 581–601. doi: 10.1007/s11103-009-9493-y 19449183

[B32] Gibalová A. Steinbachová L. Hafidh S. Bláhová V. Gadiou Z. Michailidis C.. (2017). Characterization of pollen-expressed bZIP protein interactions and the role of ATbZIP18 in the male gametophyte. Plant Reprod. 30, 1–17. doi: 10.1007/s00497-016-0295-5 27896439

[B33] Giri M. K. Singh N. Banday Z. Z. Singh V. Ram H. Singh D.. (2017). GBF1 differentially regulates CAT2 and PAD4 transcription to promote pathogen defense in arabidopsis thaliana. Plant J. 91, 802–815. doi: 10.1111/tpj.13608 28622438

[B34] Gustafson-Brown C. Savidge B. Yanofsky M. F. (1994). Regulation of the arabidopsis floral homeotic gene APETALA1. Cell. 76, 131–143. doi: 10.1016/0092-8674(94)90178-3 7506995

[B35] Hahlbrock K. Scheel D. (1989). Physiology and molecular biology of phenylpropanoid metabolism. Annu. Rev. Plant Physiol. Plant Mol. Biol. 40, 347–369. doi: 10.1146/annurev.pp.40.060189.002023

[B36] Hanano S. Goto K. (2011). Arabidopsis TERMINAL FLOWER1 is involved in the regulation of flowering time and inflorescence development through transcriptional repression. Plant Cell. 23, 3172–3184. doi: 10.1105/tpc.111.088641 21890645 PMC3203435

[B37] Hartmann L. Pedrotti L. Weiste C. Fekete A. Schierstaedt J. Göttler J.. (2015). Crosstalk between two bZIP signaling pathways orchestrates salt-induced metabolic reprogramming in arabidopsis roots. Plant Cell. 27, 2244–2260. doi: 10.1105/tpc.15.00163 26276836 PMC4568499

[B38] Heatherly L. G. Smith J. R. (2004). Effect of soybean stem growth habit on height and node number after beginning bloom in the mid-southern USA. Crop Sci. 44, 1855–1858. doi: 10.2135/cropsci2004.1855

[B39] Holm M. Ma L. G. Qu L. J. Deng X. W. (2002). Two interacting bZIP proteins are direct targets of COP1-mediated control of light dependent gene expression in arabidopsis. Genes Dev. 16, 1247–1259. doi: 10.1101/gad.969702 12023303 PMC186273

[B40] Howell S. H. (2013). Endoplasmic reticulum stress responses in plants. Annu. Rev. Plant Biol. 64, 477–499. doi: 10.1146/annurev-arplant-050312-120053 23330794

[B41] Hu D. Li X. Yang Z. Liu S. Hao D. Chao M.. (2022). Downregulation of a gibberellin 3b-hydroxylase enhances photosynthesis and increases seed yield in soybean. New Phytol. 235, 502–517. doi: 10.1111/nph.18153 35396723

[B42] Huang L. Zhang H. Zhang H. Deng X. W. Wei N. (2015). HY5 regulates nitrite reductase 1 (NIR1) and ammonium transporter1;2 (AMT1;2) in arabidopsis seedlings. Plant Sci. 238, 330–339. doi: 10.1016/j.plantsci.2015.05.004 26259199 PMC4719586

[B43] Hurst H. C. (1995). Transcription factors 1: bZIP proteins. Protein Profile 2, 101–168. 7780801

[B44] Iwata Y. Koizumi N. (2005). An arabidopsis transcription factor, AtbZIP60, regulates the endoplasmic reticulum stress response in a manner unique to plants. Proc. Natl. Acad. Sci. U.S.A. 102, 5280–5285. doi: 10.1073/pnas.0408941102 15781873 PMC555978

[B45] Jakoby M. Weisshaar B. Droge Laser W. Vicente Carbajosa J. Tiedemann J. Kroj T.. (2002). bZIP transcription factors in arabidopsis. Trends Plant Sci. 7, 106–111. doi: 10.1016/S1360-1385(01)02223-3 11906833

[B46] Ji H. Xiao R. Lyu X. Chen J. Zhang X. Wang Z.. (2021). Differential light-dependent regulation of soybean nodulation by papilionoid-specific HY5 homologs, curr. Biol. 32, 1–13. 10.1016/j.cub.2021.12.041 35081330

[B47] Jo L. Pelletier J. M. Harada J. J. (2019). Central role of the LEAFY COTYLEDON1 transcription factor in seed development. J. Integr. Plant Biol. 61, 564–580. doi: 10.1111/jipb.12806 30916433

[B48] Jo L. Pelletier J. M. Hsu S. W. Badena R. Goldberg R. B. Harada J. J. (2020). Combinatorial interactions of the LEC1 transcription factor specify diverse developmental programs during soybean seed development. Proc. Natl. Acad. Sci. U.S.A. 117 (2), 1223–1232. doi: 10.1073/pnas.1918441117 31892538 PMC6969526

[B49] Kaminaka H. Nake C. Epple P. Dittgen J. Schutze K. Chaban C.. (2006). bZIP10-LSD1 antagonism modulates basal defense and cell death in arabidopsis following infection. EMBO J. 25, 4400–4411. doi: 10.1038/sj.emboj.7601312 16957775 PMC1570446

[B50] Kim J. C. Lee S. H. Cheong Y. H. Cheol-Min Yoo C. M. Lee S. I. Chun H. J.. (2001). A novel cold-inducible zinc finger protein from soybean, SCOF-1, enhances cold tolerance in transgenic plants. Plant J. 25 (3), 247–259. doi: 10.1046/j.1365-313x.2001.00947.x 11208017

[B51] Kuhlmann M. Horvay K. Strathmann A. Heinekamp T. Fischer U. Böttner S.. (2003). The a-helical D1 domain of the tobacco bZIP transcription factor BZI-1 interacts with the ankyrin-repeat protein ANK1 and is important for BZI-1 function, both in auxin signaling and pathogen response. J. Biol. Chem. 278, 8786–8794. doi: 10.1074/jbc.M210292200 12499372

[B52] Lara P. Onate-Sanchez L. Abraham Z. Ferrandiz C. Diaz I. Carbonero P.. (2003). Synergistic activation of seed storage protein gene expression in arabidopsis by ABI3 and two bZIPs related to OPAQUE2. J. Biol. Chem. 278, 21003–21011. doi: 10.1074/jbc.M210538200 12657652

[B53] Li B. Liu Y. Cui X. Y. Fu J. D. Zhou Y. B. Zheng W. J. (2019). Genome-wide characterization and expression analysis of soybean tga transcription factors identified a novel tga gene involved in drought and salt tolerance. Front. Plant Sci. 10, 549. 31156656 10.3389/fpls.2019.00549 PMC6531876

[B54] Li Y. Chen Q. Nan H. Li X. Lu S. Zhao X.. (2017). Overexpression of GmFDL19 enhances tolerance to drought and salt stresses in soybean. PloS One 12 (6), e0179554. doi: 10.1371/journal.pone.0179554 28640834 PMC5480881

[B55] Li X. Fang C. Yang Y. Lv T. Su T. Chen L.. (2021). Overcoming the genetic compensation response of soybean florigens to improve adaptation and yield at low latitudes, curr. Biol. 31, 1–13. doi: 10.1016/j.cub.2021.12.041 34270946

[B56] Li D. Fu F. Zhang H. Song F. (2015). Genome-wide systematic characterization of the bZIP transcriptional factor family in tomato (Solanum lycopersicum l.). BMC Genom. 16, 771. doi: 10.1186/s12864-015-1990-6 PMC4603586 26459863

[B57] Liao Y. Zou H. F. Wei W. Hao Y. J. Tian A. G. Huang J.. (2008). Soybean GmbZIP44, GmbZIP62 and GmbZIP78 genes function as negative regulator of ABA signaling and confer salt and freezing tolerance in transgenic arabidopsis. Planta. 228, 225–240. doi: 10.1007/s00425-008-0731-3 18365246

[B58] Liu B. Watanabe S. Uchiyama T. Kong F. Kanazawa A. Xia Z.. (2010). The soybean stem growth habit gene Dt1 is an ortholog of arabidopsis TERMINAL FLOWER1. Plant Physiol. 153, 198–210. doi: 10.1104/pp.109.150607 20219831 PMC2862436

[B59] Lopez-Molina L. Mongrand S. Chua N. H. (2001). A postgermination developmental arrest checkpoint is mediated by abscisic acid and requires the ABI5 transcription factor in arabidopsis. Proc. Natl. Acad. Sci. U.S.A. 98, 4782–4787. doi: 10.1073/pnas.081594298 11287670 PMC31911

[B60] Lozano-Sotomayor P. Chávez Montes R. A. Silvestre-Vañó M. Herrera-Ubaldo H. Greco R. Pablo-Villa J.. (2016). Altered expression of the bZIP transcription factor DRINK ME affects growth and reproductive development in arabidopsis thaliana. Plant J. 88, 437–451. doi: 10.1111/tpj.13264 27402171

[B61] Lu S. J. Dong L. D. Fang C. Liu S. L. Kong L. P. Cheng Q.. (2020). Stepwise selection on homeologous PRR genes controlling flowering and maturity during soybean domestication. Nat. Genet. 52, 428–436. doi: 10.1038/s41588-020-0604-7 32231277

[B62] Lu S. J. Zhao X. H. Hu Y. L. Liu S. L. Nan H. Y. Li X. M.. (2017). Natural variation at the soybean J locus improves adaptation to the tropics and enhances yield. Nat. Genet. 49, 773–779. doi: 10.1038/ng.3819 28319089

[B63] Lyu X. Cheng Q. Qin C. Li Y. Xu X. Ji R.. (2021). GmCRY1s modulate gibberellin metabolism to regulate soybean shade avoidance in response to reduced blue light. Mol. Plant 14, 298–314. doi: 10.1016/j.molp.2020.11.016 33249237

[B64] Maier A. T. Stehling-sun S. Offenburger S. Lohmann J. U. (2011). The bZIP transcription factor PERIANTHIA: a multifunctional hub for meristem control. Front. Plant Sci. 2, 1–17. doi: 10.3389/fpls.2011.00079 22645551 PMC3355747

[B65] Manavalan L. P. Guttikonda S. K. Tran L. P. Nguyen H. T. (2009). Physiological and molecular approaches to improve drought resistance in soybean. Plant Cell Physiol. 50, 1260–1276. doi: 10.1093/pcp/pcp082 19546148

[B66] Manavalan L. P. Prince S. J. Musket T. A. Chaky J. Deshmukh R. Vuong T. D.. (2015). Identification of novel QTL governing root architectural traits in an interspecific soybean population. PloS One 10 (3), e0120490. doi: 10.1371/journal.pone.0120490 25756528 PMC4355624

[B67] Maurya J. P. Sethi V. Gangappa S. N. Gupta N. Chattopadhyay S. (2015). Interaction of MYC2 and GBF1 results in functional antagonism in blue light-mediated arabidopsis seedling development. Plant J. 83, 439–450. doi: 10.1111/tpj.12899 26047210

[B68] Miao Z. H. Liu X. Lam E. (1994). TGA3 is a distinct member of the TGA family of bZIP transcription factors in arabidopsis thaliana. Plant Mol. Biol. 25, 1–11. doi: 10.1007/BF00024193 8003690

[B69] Müller M. J. Berger S. (2008). General detoxification and stress responses are mediated by oxidized lipids through TGA transcription factors in arabidopsis. Plant Cell. 20, 768–785. doi: 10.1105/tpc.107.054809 18334669 PMC2329937

[B70] Murmu J. Bush M. J. DeLong C. Li S. Xu M. Khan M.. (2010). Arabidopsis basic leucine-zipper transcription factors TGA9 and TGA10 interact with floral glutaredoxins ROXY1 and ROXY2 and are redundantly required for anther development. Plant Physiol. 154, 1492–1504. doi: 10.1104/pp.110.159111 20805327 PMC2971623

[B71] Nan H. Cao D. Zhang D. Li Y. Lu S. Tang L.. (2014). GmFT2a and GmFT5a redundantly and differentially regulate flowering through interaction with and upregulation of the bZIP transcription factor GmFDL19 in soybean. PloS One 9, e97669. doi: 10.1371/journal.pone.0097669 24845624 PMC4028237

[B72] Oyama T. Shimura Y. Okada K. (1997). The arabidopsis HY5 gene encodes a bZIP protein that regulates stimulus-induced development of root and hypocotyl. Genes Dev. 11, 2983–2995. doi: 10.1101/gad.11.22.2983 9367981 PMC316701

[B73] Patil V. S. Wuike R. V. Thakare C. S. Chirame B. B. (1997). Viability of uredospores of phakopsora pachyrhizi syd. at different storage conditions. J. Maharashtra Agric. Univ. 22, 260–261.

[B74] Pedersen P. Lauer J. G. (2004). Response of soybean yield components to management system and planting date. Agron. J. 96, 1372–1381. doi: 10.2134/agronj2004.1372

[B75] Price A. H. Townend J. Jones M. P. Audebert A. Courtois B. (2002). Mapping QTLs associated with drought avoidance in upland rice grown in the Philippines and West Africa. Plant Mol. Biol. 48, 683–695. doi: 10.1023/A:1014805625790 11999843

[B76] Schindler U. Menkens A. E. Beckmann H. Ecker J. R. Cashmore A. R. (1992). Heterodimerization between light-regulated and ubiquitously expressed arabidopsis GBF bZIP proteins. EMBO J. 11, 1261–1273. doi: 10.1002/j.1460-2075.1992.tb05170.x 1373374 PMC556574

[B77] Schmid M. Davison T. S. Henz S. R. Pape U. J. Demar M. Vingron M.. (2005). A gene expression map of arabidopsis thaliana development. Nat. Genet. 37 (5), 501–506. doi: 10.1038/ng1543 15806101

[B78] Schmutz J. Cannon S. Schlueter J. Ma J. Mitros T. Nelson W.. (2010). Genome sequence of the palaeopolyploid soybean. Nature 463, 178–183. doi: 10.1038/nature08670 20075913

[B79] Shen L. Liu Z. Yang S. Yang T. Liang J. Wen J.. (2016). Pepper CabZIP63 acts as a positive regulator during ralstonia solanacearum or high temperature-high humidity challenge in a positive feedback loop with CaWRKY40. J. Exp. Bot. 67, 2439–2451. doi: 10.1093/jxb/erw069 26936828 PMC4809298

[B80] Shin S. Y. Kim S. H. Kim H. J. Jeon S. J. Sim S. A. Ryu G. R.. (2016). Isolation of three b-box zinc finger proteins that interact with STF1 and COP1 defines a HY5/COP1 interaction network involved in light control of development in soybean. BBRC. 478, 1080e1086. doi: 10.1016/j.bbrc.2016.08.069 27524234

[B81] Skubacz A. Daszkowska-Golec A. Szarejko I. (2016). The role and regulation of ABI5 (ABA-insensitive 5) in plant development, abiotic stress responses and phytohormone crosstalk. Front. Plant Sci. 7, 1884. doi: 10.3389/fpls.2016.01884 28018412 PMC5159420

[B82] Smith T. J. Camper H. M. (1970). Effects of seed size on soybean performance. Agro. J. 67, 681–684. doi: 10.2134/agronj1975.00021962006700050025x

[B83] Smykowski A. Zimmermann P. Zentgraf U. (2010). G-Box binding factor1 reduces CATALASE2 expression and regulates the onset of leaf senescence in arabidopsis. Plant Physiol. 153, 1321–1331. doi: 10.1104/pp.110.157180 20484024 PMC2899923

[B84] Song Y. H. Yoo C. M. Hong A. P. Kim S. H. Jeong H. J. Kim H. J.. (2008). DNA-Binding study identifies c-box and hybrid C/G-box or C/A-box motifs as high-affinity binding sites for STF1 and LONG HYPOCOTYL5 proteins. Plant Physiol. 146, 1862e1877. doi: 10.1104/pp.107.113217 18287490 PMC2287355

[B85] Sun T. Busta L. Zhang Q. Ding P. Jetter R. Zhang Y. (2018). T: GACG-binding factor 1 (TGA1) and TGA4 regulate salicylic acid and pipecolic acid biosynthesis by modulating the expression of systemic acquired resistance deficient 1 (SARD1) and calmodulin-binding protein 60 g (CBP60 g). New Phytol. 217, 344–354. doi: 10.1111/nph.14780 28898429

[B86] Sun X. Li Y. Cai H. Bai X. Ji W. Ding X.. (2012). The arabidopsis AtbZIP1 transcription factor is a positive regulator of plant tolerance to salt, osmotic and drought stresses. J. Plant Res. 125, 429–438. doi: 10.1007/s10265-011-0448-4 21938515

[B87] Sussmilch F. C. Berbel A. Hecht V. Vander Schoor J. K. Ferrándiz C. Madueño F.. (2015). Pea VEGETATIVE2 is an FD homolog that is essential for flowering and compound inflorescence development. Plant Cell. 27 (4), 1046–1046. doi: 10.1105/tpc.115.136150 25804541 PMC4558695

[B88] Takeshima R. Nan H. Harigai K. Dong L. Zhu J. Lu S.. (2019). Functional divergence between soybean FLOWERING LOCUS T orthologues FT2a and FT5a in post-flowering stem growth. J. Exp. Bot. 70, 3941–3953. doi: 10.1093/jxb/erz199 31035293 PMC6685666

[B89] Tian Z. Wang X. Lee R. Li Y. Specht J. E. Nelson R. L.. (2010). Artificial selection for determinate growth habit in soybean. P. Natl. Acad. Sci. U.S.A. 107, 8563–8568. doi: 10.1073/pnas.1000088107 PMC2889302 20421496

[B90] Uno Y. Furihata T. Abe H. Yoshida R. Shinozaki K. Yamaguchi-Shinozake K. (2000). Arabidopsis basic leucine zipper transcription factors involved in an abscisic acid-dependent signal transduction pathway under drought and high-salinity conditions. P. Natl. Acad. Sci. U.S.A. 97 (21), 11632–11637. doi: 10.1073/pnas.190309197 PMC17252 11005831

[B91] Van Leene J. Blomme J. Kulkarni S. R. Cannoot B. De Winne N. Eeckhout D.. (2016). Functional characterization of the arabidopsis transcription factor bZIP29 reveals its role in leaf and root development. J. Exp. Bot. 67, 5825–5840. doi: 10.1093/jxb/erw347 27660483 PMC5066499

[B92] Vitale A. Ceriotti A. (2004). Protein quality control mechanisms and protein storage in the endoplasmic reticulum. a conflict of interests? Plant Physiol. 136, 3420–3426. 15542495 10.1104/pp.104.050351 PMC527140

[B93] Wang Z. Cheng K. Wan L. Yan L. Jiang H. Liu S.. (2015). Genome-wide analysis of the basic leucine zipper (bZIP) transcription factor gene family in six legume genomes. BMC Genom. 16, 1053. doi: 10.1186/s12864-015-2258-x PMC4676100 26651343

[B94] Wang L. Fobert P. R. (2013). Arabidopsis clade I TGA factors regulate apoplastic defences against the bacterial pathogen pseudomonas syringae through endoplasmic reticulum- based processes. PloS One 8, e77378. doi: 10.1371/journal.pone.0077378 24086773 PMC3785447

[B95] Wang T. Guo J. Peng Y. Lyu X. Liu B. Sun S.. (2021). Light-induced mobile factors from shoots regulate rhizobium-triggered soybean root nodulation. Science. 374, 65–71. doi: 10.1126/science.abh2890 34591638

[B96] Wei K. Chen J. Wang Y. Chen Y. Chen S. Lin Y.. (2012). Genome-wide analysis of bZIP-encoding genes in maize. DNA Res. 19, 463–476. doi: 10.1093/dnares/dss026 23103471 PMC3514857

[B97] Wei L. Q. Xu W. Y. Deng Z. Y. Su Z. Xue Y. Wang T. (2010). Genome-scale analysis and comparison of gene expression profiles in developing and germinated pollen in oryza sativa. BMC Genom. 11, 338. doi: 10.1186/1471-2164-11-338 PMC2895629 20507633

[B98] Weisshaar B. Armstrong G. A. Block A. da Costa e Silva O. Hahlbrock K. (1991). Light-inducible and constitutively expressed DNA-binding proteins recognizing a plant promoter element with functional relevance in light responsiveness. EMBO J. 10, 1777–1786. doi: 10.1002/j.1460-2075.1991.tb07702.x 2050115 PMC452849

[B99] Weltmeier F. Ehlert A. Mayer C. S. Kietrich K. Wang X. Schütze K.. (2006). Combinatorial control of arabidopsis proline dehydrogenase transcription by specific heterodimerisation of bZIP transcription factors. EMBO J. 25, 3133–3143. doi: 10.1038/sj.emboj.7601206 16810321 PMC1500977

[B100] Weltmeier F. Rahmani F. Ehlert A. Dietrich K. Schutze K. Wang X.. (2009). Expression patterns within the arabidopsis C/S1 bZIP transcription factor network: availability of heterodimerization partners controls gene expression during stress response and development. Plant Mol. Biol. 69, 107–119. doi: 10.1007/s11103-008-9410-9 18841482 PMC2709229

[B101] Wigge P. A. Kim M. C. Jaeger K. E. Busch W. Schmid M. Lohmann J. U.. (2005). Integration of spatial and temporal information during floral induction in arabidopsis. Science. 309, 1056–1059. doi: 10.1126/science.1114358 16099980

[B102] Wingender E. Chen X. Fricke E. Geffers R. Hehl R. Liebich I.. (2001). The TRANSFAC system on gene expression regulation. Nucleic Acids Res. 29, 281–283. doi: 10.1093/nar/29.1.281 11125113 PMC29801

[B103] Wingender R. Rohrig H. Horicke C. Schell J. (1990). Cis-regulatory elements involved in ultraviolet light regulation and plant defense. Plant Cell. 2, 1019–1026. 2136625 10.1105/tpc.2.10.1019 PMC159950

[B104] Xiang C. Miao Z. Lam E. (1997). DNA-Binding properties, genomic organization and expression pattern of TGA6, a new member of the TGA family of bZIP transcription factors in arabidopsis thaliana. Plant Mol. Biol. 34, 403–415. doi: 10.1023/A:1005873500238 9225852

[B105] Xu Z. Ali Z. Xu L. He X. Huang H. Yi J.. (2016). The nuclear protein GmbZIP110 has transcription activation activity and plays important roles in the response to salinity stress in soybean. Sci. Rep. 6, 20366. doi: 10.1038/srep20366 26837841 PMC4738249

[B106] Xu C. Cao H. Zhang Q. Wang H. Xin W. Xu E.. (2018). Control of auxin-induced callus formation by bZIP59-LBD complex in arabidopsis regeneration. Nat. Plants. 4, 108–115. doi: 10.1038/s41477-017-0095-4 29358751

[B107] Xu L. Xu Z. Liu X. Huang Y. He X. Ma H.. (2015). The subcellular localization and ectopic expression analysis in arabidopsis of soybean GmbZIP60 gene. J. Plant Biochem. Biotechnol. 24 (1), 9–17. doi: 10.1007/s13562-013-0228-4

[B108] Yanagisawa S. (2014). Transcription factors involved in controlling the expression of nitrate reductase genes in higher plants. Plant Sci. 229, 167–171. doi: 10.1016/j.plantsci.2014.09.006 25443843

[B109] Yang O. Popova O. V. Süthoff U. Lüking I. Dietz K. J. Golldack D. (2009). The arabidopsis basic leucine zipper transcription factor AtbZIP24 regulates complex transcriptional networks involved in abiotic stress resistance. Gene. 436, 45–55. doi: 10.1016/j.gene.2009.02.010 19248824

[B110] Yang Y. Yu T. F. Ma J. Chen J. Zhou Y. B. Chen M.. (2020). The soybean bZIP transcription factor gene GmbZIP2 confers drought and salt resistances in transgenic plants. Int. J. Mol. Sci. 21, 670. doi: 10.3390/ijms21020670 31968543 PMC7013997

[B111] Yoshida T. Fujita Y. Maruyama K. Mogami J. Todaka D. Shinozaki K.. (2015a). Four arabidopsis AREB/ABF transcription factors function predominantly in gene expression downstream of SnRK2 kinases in abscisic acid signalling in response to osmotic stress. Plant Cell Environ. 38 (1), 35–49. doi: 10.1111/pce.12351 24738645 PMC4302978

[B112] Yoshida T. Mogami J. Yamaguchi-Shinozaki K. (2015b). Omics approaches toward defining the comprehensive abscisic acid signaling network in plants. Plant Cell Physiol. 56, 1043–1052. doi: 10.1093/pcp/pcv060 25917608

[B113] Yoshida K. Wakamatsu S. Sakuta M. (2008). Characterization of SBZ1, a soybean bZIP protein that binds to the chalcone synthase gene promoter. Plant Biotechnol. 25, 131–140. doi: 10.5511/plantbiotechnology.25.131

[B114] Yue L. Li X. Fang C. Chen L. Yang H. Yang J.. (2021). FT5a interferes with the Dt1-AP1 feed-back loop to control flowering time and shoot determinacy in soybean. J. Integr. Plant Biol. 63, 1004–1020. doi: 10.1111/jipb.13070 33458938

[B115] Zhang M. Liu Y. Cai H. Guo M. Chai M. She Z.. (2020). The bZIP transcription factor GmbZIP15 negatively regulates salt- and drought-stress responses in soybean. Int. J. Mol. Sci. 21, 7778. doi: 10.3390/ijms21207778 33096644 PMC7589023

[B116] Zhang M. Liu Y. Shi H. Guo M. Chai M. He Q.. (2018). Evolutionary and expression analyses of soybean basic leucine zipper transcription factor family. BMC Genom. 19, 159. doi: 10.1186/s12864-018-4511-6 PMC5824455 29471787

